# An APOC3 3′UTR variant associated with plasma triglycerides levels and coronary heart disease by creating a functional miR-4271 binding site

**DOI:** 10.1038/srep32700

**Published:** 2016-09-14

**Authors:** Sen-Lin Hu, Guang-Lin Cui, Jin Huang, Jian-Gang Jiang, Dao-Wen Wang

**Affiliations:** 1 Institute of Hypertension and Department of Internal Medicine, Division of Cardiology, Tongji Hospital, Tongji Medical College, Huazhong University of Science and Technology, Wuhan 430030, China

## Abstract

Apolipoprotein C-III (APOC3) is a key regulator of plasma triglycerides levels. Increasing evidence has shown that loss-of-function mutations in APOC3 is associated with reduction in plasma triglycerides levels and will confer a benefit in patients at high risk for cardiovascular disease. However, these favorable mutations were extremely distribution discrepant among different ethnics. In this study, the APOC3 gene was resequenced and we identified a common variant which located in the microRNA-binding site in APOC3 and would affect its expression and the risk of coronary heart disease (CHD). The molecular mechanism was explored. We found that the T allele of rs4225 suppressed APOC3 translation by facilitating miR-4271 binding, but not the G allele. Subjects carrying the GG genotype had higher plasma APOC3 levels (p for trend = 0.03) than those with the TT genotype. Furthermore, the T allele was significantly associated with decreased triglyceride levels [Beta (SE): −0.024 (0.020), P = 0.03]. Finally, the case-control study suggested that the TT genotype resulted in a significant reduction in overall CHD risk [OR, 0.89 (95% confidence interval, 0.77–0.98), P = 0.009]. In conclusion, our results provide evidence that the rs4225 in the 3′-UTR of APOC3 might contribute to the risk of CHD by interfering with miR-4271 binding.

Hypertriglyceridemia (HTG) is a common metabolic disease resulting from complex interactions between genetic and environmental factors^1^,^2^. Recent evidence have shown a long-standing association existed between elevated triglycerides levels and cardiovascular disease (CVD)^3^,^4^. Heritability accounts for more than 50% of the individual variation in triglycerides levels^5^. At present, genome wide association studies have identified common DNA sequence variants at more than 50 genetic loci that are related to plasma triglycerides^6^,^7^. Deleterious mutations in these genes cause HTG and various SNPs have been associated with both mild and severe HTG. They may directly or indirectly promote CVD^8^,^9^.

Apolipoprotein C-III is an important component of triglyceride-rich lipoproteins whose normal function is to inhibit hydrolysis of these lipoproteins in plasma and thereby preserves triglycerides levels^10^. Several polymorphisms in the APOC3 gene have been found in association with HTG, but the link with CHD risk between ethnics was still controversial^5^,^11^,^12^,^13^. Among them, two functional polymorphisms T-455C and C-482T which located in insulin-responsive element and insulin-regulating region were thought to exert their action by down-regulating APOC3 gene expression. But a lack of association was detected between these polymorphisms and risk of CHD^13^. A recent research hotspot: null APOC3 variant, R19X, found to be associated with high-density lipoprotein (HDL) and triglycerides (TG) levels has a cardioprotective effect^5^,^14^,^15^. However, increasing evidence have shown this mutation in the Amish was to be population-specific as it was not detected in non-Amish individuals of European descent given available data at the time^14^,^16^. Recently, the TG and HDL Working Group of the Exome Sequencing Project found rare APOC3 variants that were associated with a 44% reduction in plasma TG levels. In a cohort of 75,725 Danes, carriers of these variants had a 41% reduction in risk of CHD^5^. However, none of the loss-of-function mutations reported in European and African individuals were observed in the Chinese Han population^17^.

Taken together, although more and more findings have provided compelling evidence that reducing APOC3 expression will reduce CHD risk, the question remains as to whether the reduced CHD risk in APOC3 variant carriers is due to lower plasma TG levels or to other associated factors (APOC3, or remnant lipoproteins, lower plasma levels of LDL cholesterol (LDL-C), or increased levels of HDL-C)^15^. Currently, studies on genetic variants of APOC3 worldwide are mainly focused on the exome sequencing, in which the TG-related genetic changes have been thoroughly investigated. However, protein-coding genes account for only a very small proportion of the transcribed human genome, thus highlighting a lack of understanding of the possible contributions of noncoding RNAs to human traits and disease.

Therefore, the mechanism exploration of other functional common or rare variants involved in TG regulation may lead to a breakthrough. Through a bioinformatics approach, we identified a variant, rs4225, which located in the miRNA-4271 target site in the 3′-untranslated region (3′-UTR) of APOC3. To our knowledge, the role of APOC3 polymorphisms in regulation of plasma lipids has thus far not been investigated. Herein, we hypothesize that variant rs4225 could modify miR-4271 binding to APOC3 and to interfere APOC3 concentration thus contribute to the genetic susceptibility to cardioprotective effect.

## Results

### DNA resequencing results

In this study, a total of 400 unrelated Chinese participants were sequenced and our results demonstrated that only synonymous and intronic variants without functional consequences were identified. Consolidated with our previously reported resequencing data^18^, a total of 800 subjects’ sequencing data were reanalyzed and none of the loss-of-function mutations reported in European and African individuals were observed in the current cohort (Table 1).

### Effects of polymorphisms on *in vitro* activity of the APOC3 3′UTR

**Figure 1:**
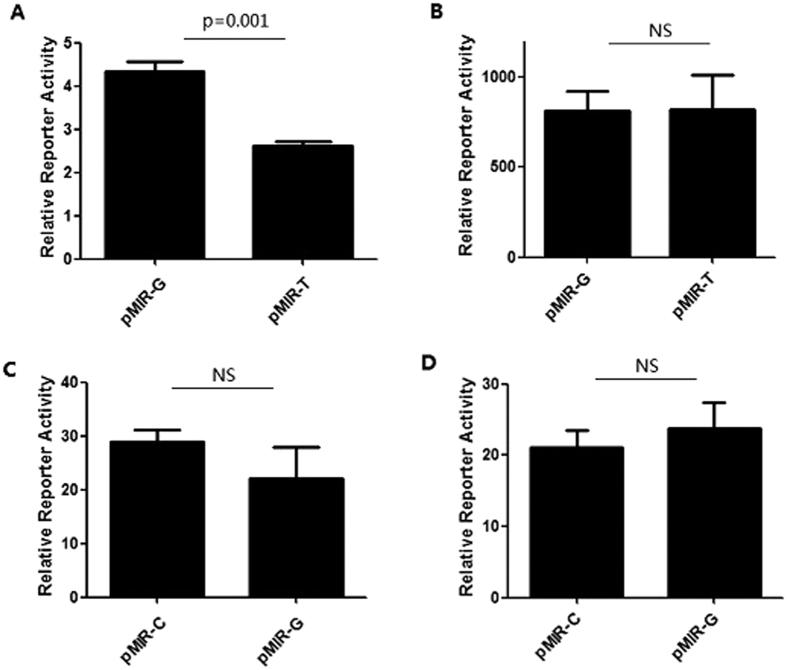
Expression studies of APOC3 3′UTR constructs carrying rs4225G/T (**A,B**) and rs5128C/G (**C,D**) alleles in HepG2 cells (**A,D**) or 293Tcells (**B,C**), respectively. APOC3 3′UTR activity is expressed as fold change of Relative Luciferase to pMIR basic. Mutant construct was compared with the wild-type construct for each comparison. Values are mean  ±  SE of three independent experiments each corresponding to at least six replicates.

Using the genotypes of 384 healthy controls, we defined the haploblock structure of SNPs within the region of APOC3 gene in the Han Chinese population (Supplemental Figure 1). There are two SNPs (rs5128 and rs4225) located in the 3′-untranslated region (3′-UTR) of APOC3, which may function as regulatory SNPs to affect the phenotypes and disease susceptibility. To test whether the polymorphisms in the 3′-UTR of APOC3 were functionally important for the regulation of APOC3 expression, we performed functional analyses comparing the activities of two SNPs. As shown in Fig. 1, reporter gene expression of the pMIR-T (with the T allele of rs4225) allele in HepG2 cells was significantly reduced compared with the pMIR-G (with the G allele of rs4225) allele (34 ± 1.32%, P = 0.001) (Fig. 1A). However, this effect was not significant in the 293T cells (Fig. 1B) and no effect on luciferase activity was observed in rs5128 luciferase assays both in 293T cells and HepG2 cells (Fig. 1C,D). These results indicate that endogenous hepatic regulator factor may be able to target the APOC3 3′ UTR with the rs4225-T allele and decrease APOC3 3′ UTR luciferase expression.

### Variant rs4225 of APOC3 is in the miR-4271 binding site

Given that rs4225 was located in the conserved region of APOC3 (UCSC Genome Browser; http://genome.ucsc.edu/) and in strong LD with rs5128 in the Han Chinese population (D′ = 1.0) (Supplementary Figure 1) and had important clinical relevance, we focused on rs4225 for further functional analysis.

**Figure 2:**
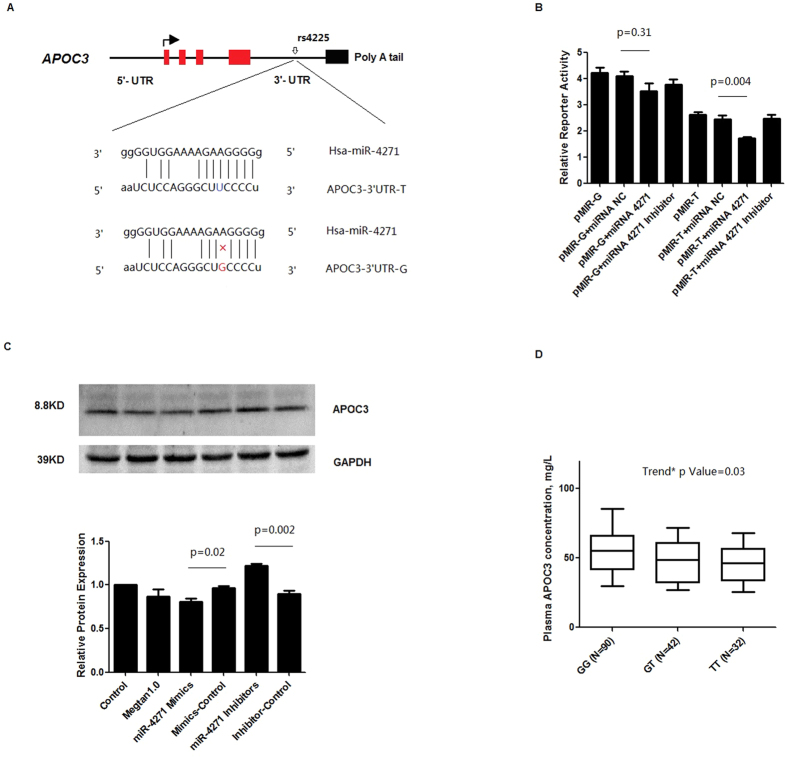
Functional validation of the miR-4271 binding site in the APOC3 3′UTR and the influence of the SNP rs4225. (**A**) APOC3 gene structure and the rs4225 polymorphism in the 3′ UTR at the miR-4271-binding site. The polymorphism rs4225 is a G to T change (mRNA sequence as reference) located in the predicted binding site for miR-4271 in the 3′-UTR of the APOC3 gene. At rs4225, allele T base-paired with U in Watson–Crick mode (shown with a solid line), whereas allele G did not (shown without line). (**B**) The interaction between miR-4271 and APOC3 using a reporter gene assay in HepG2 cells. Luciferase plasmid contains pMIR-T or pMIR-G was cotransfected with negative control miRNA (miR-NC) or miR-4271. For each transfection, at least six replicate assays were performed. Luciferase activity was normalized by Renilla luciferase activity for each sample. (**C**) miR-4271 negatively regulates APOC3 through binding to 3′-UTR of the APOC3. Inhibiting the expression of hsa-miR-4271 increased APOC3 expression in HepG2 cells analyzed by western blotting. Columns, mean of three independent experiments; bars, SE. (**D**) The plasma levels of APOC3 with different genotypes of the polymorphism rs4225. Individuals with TT genotype have lower plasma APOC3 levels vs. GG genotypes (p for trend = 0.03), and the data are presented as box (25th percentile, median and 75th percentile) and whisker (10th and 90th percentiles) plots.

Further bioinformatics analysis by computer alignment demonstrated that a nucleotide at position rs4225 located in the miRNA-4271 seed binding site (Fig. 2A) which is conservative in humans (UCSC Genome Browser; http://genome.ucsc.edu/).

To test the prediction model that miR-4271 can functionally interact with the 3′-UTR of APOC3, luciferase expression vectors containing the G or T allele (pMIR-G or pMIR-T, respectively) were constructed for functional assessment of the binding of miRNA with the mutant APOC3 3′UTR. HepG2 cells were cotransfected with pMIR-G or pMIR-T and miRNA precursor (miR-4271), or control mimic (miR-NC). Compared with control miRNA, the APOC3 3′UTR containing the T allele showed a significant decrease in luciferase activity in the presence of miR-4271 (−36 ± 1.22% p = 0.004) (Fig. 2B). However, the 3′UTR containing the G allele showed nonsignificant increase in the level of luciferase activity in the presence of miR-4271 compare to control miRNA (p = 0.31). The similar results were observed in 293T cells (Supplementary Figure 2).

**Figure 3:**
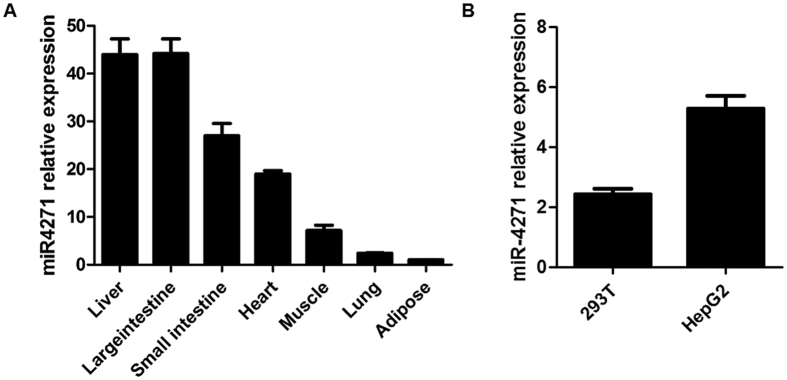
Comparision of real-time quantitative PCR for miR-4271 expression in different human tissues (**A**) or human cell lines including HepG2 and 293T (**B**). Data are means ± (SE) from three independent experiments analyzed in six replicates. Data are normalized with reference microRNAs, as mentioned in the text.

In order to further confirm the effects of miR-4271 on endogenous APOC3 expression, we sequenced nine of the human hepatoma cell lines (including HepG2, HuH-7, Hep3B, LM3, HLE, HLF, 97L, PLC, L02, etc.) and HepG2 was identified to be TT genotype, while 97L was identified to be GG genotype (Supplementary Figure 3). Next we used these cell lines to do experiments. Western blot results showed that miR-4271 downregulated APOC3 expression and the inhibition of miR-4271 expression using its inhibitor significantly upregulated expression of APOC3 in HepG2 cells (Fig. 2C). No significant effect of miR-4271 on APOC3 expression was found in 97L cells (Supplementary Figure 4). Moreover, miR-4271 was expressed in multiple human tissues and hepatic cell lines and was most highly expressed in human hepatic tissue (Fig. 3). It should be noted that the expression level of miR-4271 is higher in HepG2 cells than that in 293T cells (Fig. 3) and this may partly explain the difference of luciferase expression levels of allele-specific constructs in different cells.

*In vivo*, plasma concentrations of APOC3 were lower in subjects with the TT genotype than in those with the GT or GG genotypes (p for trend = 0.03) (Fig. 2D).

### Association of common variants with lipid level

The relationship between rs4225 polymorphism and plasma lipids concentrations was subsequently analyzed in 2982 unrelated individuals. As shown in Table 2, the proportion of a 1-SD change in plasma TG levels for each copy of the risk allele were 0.09, and the contribution of this genetic effect can explain 6.2% of the total variance of plasma TG levels in our population. We did not find this polymorphism be associated with HDL-C, LDL-C or TC (Table 2).

### The G-allele of variant rs4225 is possibly associated with CHD risk

The distribution of variant rs4225 is shown in Table 3, and the frequencies of this variant did not deviate significantly from the HWE in cases and control subjects (all P > 0.05). Our results showed that rs4225-T allele was significantly associated with decreased risk of CHD in our study independent of traditional cardiovascular risk factors in recessive model (OR = 0.89, 95% CI = 0.77 to 0.98).

### Population angiographic characteristics

The CHD cases documented angiographically as having >50% diameter stenosis in at least 1 coronary artery were eligible for our analysis between the different genotypes. The angiographic characteristics between different genotypes of rs4225 were analyzed. As shown in Supplementary Table 1, there was a trend that patients with G allele demonstrated more severe angiographic CAD as indicated by the higher incidence of multivessel disease, and by a larger occurrence of LAD lesions. Conversely, the RCA lesions have higher proportion in the TT genotype compared with the GG and GT genotypes. However, this trend was not significant.

## Discussion

The main findings of this study are that the T allele of rs4225 in APOC3 3′UTR interacts with the miR-4271 binding site, therefore decreases the translation of APOC3, and the case-control study shows that rs4225T might be associated with lower CHD risk.

The hypertriglyceridemic effect of apolipoprotein C3 is attributable to its extracellular and intracellular roles in triglyceride metabolism^12^. Its normal function is to inhibit hydrolysis of the lipoproteins in plasma and thereby preserves triglycerides levels. ApoC-III plasma levels are independently associated with the progression of CHD^10^, and the level of apoC-III in VLDL and LDL is a more specific measurement of CHD risk compared with plasma TG levels^19^. Therefore, inhibiting apo-CIII is an attractive way to reduce triglycerides levels and hence reduce cardiovascular risk^20^. Recently, with the development of the “Next-generation” sequencing technology, lots of loss-of-function mutations of the APOC3 gene in some cohorts were identified and these mutations were associated with decreased triglycerides levels and a reduced risk of ischemic vascular disease^5^,^12^. This led to the hypothesis that the beneficial effect could be observed in CHD in carriers of APOC3 loss-of-function mutations. However, in our study, only APOC3 synonymous and intronic variants with no functional consequences were identified. None of the loss-of-function mutations reported in European and African individuals were observed. Therefore, the genetic background of ischemic vascular disease is highly variable among different ethnic groups.

Up to now, GWASs have been extensively used to identify common variants, termed SNPs, which were associated with various human traits. However, protein-coding genes account for only a very small proportion of the transcribed human genome, and thus, exploration of the common variants located in noncoding RNAs regulatory region may provide more underlying mechanisms to understand human traits and disease^21^. Among these common polymorphisms within APOC3 gene, previous reports have shown two functional polymorphisms T-455C and C-482T, located in insulin-responsive element and insulin-regulating region respectively, which were thought to exert their action by down-regulating APOC3 gene expression^22^. But a lack of association was detected between these polymorphisms and risk of CHD in the Han Chinese population^13^. The SNP rs5128 which is located in the 3′ UTR was previously reported significantly associated with TG levels in Chinese Han population^18^, but these associations among different studies were conflicting and inconclusive^23^. It is worth noting that, no publication reported rs5128 function in relative luciferase expression and no transcription factors mapped this site. On the contrary, we found that rs4225 T allele significantly changed relative luciferase expression. Actually, rs4225 was in a moderate LD with rs5128 in http://snpinfo.niehs.nih.gov/), but however, higher LD between above two SNPs was observed in Chinese Han population (D′ = 1.0, Supplementary Figure 1). Thus, these results may give a hypothesis that rs4225 but not rs5128 is the causal variant.

Apolipoprotein C-III is a key regulator of lipoprotein metabolism and plays a pivotal role in regulating plasma triglyceride levels^24^,^25^. Elevated APOC3 levels are an independent risk factor for cardiovascular disease, especially when APOC3 is present on apolipoprotein B–containing lipoproteins^26^,^27^. Conversely, genetic variants that result in a loss of function and attenuate levels of APOC3 in plasma are associated with a reduced risk of coronary heart disease^28^. Recently, Wagschal *et al*. reported that altered expression of miRNAs may contribute to abnormal blood lipid levels, predisposing individuals to human cardiometabolic disorders^21^. In the present study, the GG genotype (rs4225) is resistant to miR-4271-induced down regulation of APOC3, resulting in higher plasma levels of APOC3 than that in the other genotypes. These effects could be translated into a reduction of CHD risk. However, up to now the function of miR-4271 is largely unknown. Goff, L. A. *et al*. first reported that it may be expressed early in stem cell differentiation which are required for maintenance of pluripotency as well as differentiation^29^. Our data revealed that the expression pattern of APOC3 was consistent with the association of risk of CHD. This effect may be partly explained by the interaction between the miR-4271 and rs4225. However, the mechanism by which the binding of miR-4271 can regulate the plasma levels of APOC3 is not fully understood. In our study, we showed that the expression level of miR-4271 is higher in HepG2 cells than in 293T cells (Fig. 3B). This provides clues that the miR-4271 may participate in lipid metabolism in liver and its function needs to be elucidated in the future.

In the current study, we identified that the gene dosage of rs4225 were significantly related to the TG levels and the risk of CHD. Because this is an association study, we cannot rule out the presence of possible linkage disequilibrium with other neighboring genes that might explain the significant association. The CHD study was conducted in patients undergoing PCI but no replication study was performed. Therefore, our findings need to be confirmed in further larger patient populations. The negative results of angiographic characteristics were obtained from PCI patients who received no distinction of bare metal stents or drug eluting stents. These results also need a long-term follow-up analysis.

Another limitation of the present study is that the association between plasma levels of APOC3 and development of CHD has not been well established. In the Framingham Heart Study, plasma apoC-III levels were associated with plasma lipid and lipoprotein levels, and, during a median follow-up of approximately 14 years, each decrease of 1 mg/dl of plasma apoC-III was associated with a 4% decrease in the risk of incident CHD^5^.

In conclusion, our results provide evidence that the T allele of rs4225 in the 3′-UTR of APOC3 might reduce the risk of CHD, by interacting with the miR-4271, therefore decreases translation of APOC3, which would lead to decreased triglyceride levels and a reduced risk of CHD. This result might help to improve future prevention or therapy strategies for CHD.

## Methods

### Study design and eligibility

Details on sample recruitment, inclusion criteria, data collection and definition of risk factors are described in our previous report^18^. The clinical characteristics of the samples are shown in Table 4. In brief, the resequencing effort was conducted in 400 Chinese Han subjects. These controls were recruited from individuals undergoing routine health examinations at Tongji Hospital in Wuhan, Hubei province. We chose controls with maximum recorded fasting plasma triglyceride concentrations <2.3 mmol/L to exclude undiagnosed HTG^30^.

### Recruitment for the case-control samples

A total of 2627 Chinese Han CHD cases were enrolled simultaneously from hospitalized patients in Tongji Hospital and The Institute of Hypertension (Wuhan, China) between May 2004 and October 2015. The selection criteria, clinical and biochemical characteristics of the study subjects were described in detail in our previous report^31^,^32^. CHD was defined as one or more of the following diagnostic criteria: (1) >50% stenosis in at least one of the major segments of coronary arteries (the right coronary artery, left circumflex, or left anterior descending arterie) assessed by coronary angiography; (2) World Health Organization criteria for elevated cardiac enzymes (troponin T, troponin I, creatine kinase-MB, aspartate aminotransferase, and glutamic pyruvic transaminase), typical ECG changes (Minnesota Code 1.1 or 1.2 in ECG), and clinical symptoms; or (3) documented history of coronary artery bypass graft or percutaneous coronary intervention. Subjects with congenital heart disease, cardiomyopathy, valvular disease, and renal or hepatic disease were excluded from the study.

Ethnically and geographically matched controls were randomly selected from healthy residents in the community. All control subjects were free of cardiovascular diseases following the same exclusion criteria as cases. The institutional review board of Tongji hospital approved this study. Written informed consent was obtained from all participants. Experiments were conducted according to the principles expressed in the Declaration of Helsinki.

All patients and controls were carefully matched by geographic region of recruitment, were of Han Chinese ancestry, and provided written informed consent. This study was approved by the institutional ethics committees of the local participating hospitals.

### Genetic variation screening

Sequencing data of the APOC3 gene was generated by Sanger sequencing. Polymerase-chain-reaction (PCR) fragments covering the coding exons and the exon–intron boundaries (APOC3 consensus sequence NC_000011.9 GRCh37.p13) were screened using Fluorescent dye-terminator cycle and products were analyzed with an Applied Biosystems 3130xl capillary sequencer (Applied Biosystems, Foster City, CA). The Chromas program (Technelysium Pty. Ltd., Helensvale, Queensland, Australia) was used to identify putative polymorphisms that were then confirmed by two independent observers. All identified variants were confirmed by repeat sequencing. Details regarding primers are given in the online Supplemental Table 2.

### Genotyping and Bioinformatic analysis

The TaqMan SNP Genotyping Assay (Applied Biosystems) was used for SNP genotyping in this study (Supplemental Table 3). Genomic DNA was extracted from peripheral leukocytes as previously reported^32^. Probe and primer sequences for this TaqMan 5′-nuclease assay were designed by ABI Primer Expression 3.0 software and synthesized by Shanghai GeneCore BioTechnologies Co., Ltd, China. Samples were assayed along with no-template control samples, and run on an ABI 7900HT Fast Real-Time PCR System (Applied Biosystems) using the following conditions: 10 minutes at 95 °C (enzyme activation) followed by 40 cycles at 95 °C for 15 seconds and 60 °C for 1 minute (annealing/extension). The allelic discrimination results were determined after amplification by performing an endpoint read. Details of the performance for amplification reactions and the quality of genotyping were referred to in our previous report^32^. miRNA target gain and loss analysis by prediction was formed in miRNA SNP database, which is available at http://www.bioguo.org/miRNASNP/33.

### Functional analysis

Bioinformatic analysis shows that rs4225 is located at miR-4271 binding site of human APOC3 3′-UTR and therefore we tested whether rs4225*T destroyed miR-4271 binding site. p-MIR luciferase reporter containing human APOC3 3′-UTR rs4225T and rs4225G, respectively, were purchased from AuGCT Biotechnology (Beijing AuGCT Biotechnology Co.,Ltd, China) and resultant plasmids (p-MIR-T and p-MIR-G) were transformed into 293T and HepG2 cells, respectively, with or without miR-4271 to determine effects of the miR-4271 binding site by detecting fluorescence intensity according to manufacturer’s instruction.

### Luciferase assay

293T cells and HepG2 cells (1 × 10^6 ^cells per well) were co-transfected with 0.8 ug of pMIR-G or pMIR-T plasmid, 50 ng of Renilla luciferase plasmid and 100 nmol of has-miR-4271 (RIBOBIO Co., Ltd, Guangzhou, China), miRNA inhibitor(s) or miR-Negative Control (RIBOBIO Co., Ltd, Guangzhou, China), all combined with Megtran1.0 (Origene, Maryland, USA). After 48 h, the cells were washed and lysed with Passive Lysis Buffer (SIRIUS, Pforzheim, Germany). The data of luciferase expression levels were adjusted with reference to Renilla luciferase activity. Each reporter was performed six independent experiments to avoid potential experimental error.

### Determination of genotype-dependent plasma levels of APOC3

The plasma levels of APOC3 were determined in 164 samples randomly selected from controls in our study (Supplemental Table 4). To minimize the potential confounding effects, the subjects were recruited only when they also met the additional following criteria: (i) age at 35–65 years and (ii) without hyperlipidemia, cardiovascular disease or cerebrovascular diseases. The relative clinical characteristics and biochemical profiles of the samples are shown in Supplementary Table 2. The variant rs4225 was genotyped in all subjects. Among the subjects, 90 were homozygous for the G allele, 42 were heterozygous and 32 were homozygous for the T allele. Total plasma APOC3 levels were measured by means of a Human APOC3 ELISA Kit (Catalog #: ELH-ApoC3, Raybiotech, GA, USA) according to the manufacturer’s instructions. The assay sensitivity is 0.2 pg/ml, and average intra- and inter-assay coefficients of variation are 6% and 7% respectively.

### Determination of hsa-miR-4271 expression levels

Quantitative RT-PCR was used to determine the expression of miRNA hsa-miR-4271. Total RNA was extracted from human normal tissues including liver, muscle, adipose, large intestine, small intestine, heart, lung and cell lines including HepG2 and 293T cells by using Trizol Reagent Kit (Invitrogen, Carlsbad, CA) according to the manufacturer’s instructions. Human normal tissues were obtained from distal normal tissue of tumor patients. Two micrograms of total RNA were reverse-transcribed using EasyScript First-Strand cDNA Synthesis SuperMix (TransGen Biotech, Beijing, China). The miR-4271 level was quantified by real-time quantitative-PCR using Power SYBR Green PCR Master Mix (Applied Biosystems Inc.). Normalization was performed with the small nuclear RNA U6 (RNU6B; Applied Biosystems Inc.). All real-time reactions, including no-template controls and real-time minus controls, were run using the ABI 7900 Fast Real-Time PCR System (Applied Biosystems Inc.) and performed in triplicate. Relative expression was calculated using the Ct values provided by the manufacturer. This study was approved by the Review Board of Tongji Hospital and Tongji Medical College. The subjects recruited to the study provided written informed consent. The investigation conforms to the principles outlined in the Declaration of Helsinki. Tissue samples were obtained and kept frozen in liquid nitrogen and then stored at −80 °C until use.

### Western blotting analysis for APOC3

Cell transfections were performed using Megtran1.0 (Origene, Maryland, USA) according to the manufacturer’s instruction. In brief, cells grown in six-well plates were transfected with mir-4271 mimics, mir-4271 inhibitors (Supplemental Table 5) or random mir-4271 to a final concentration of 100 nM. 24 h after transfection, cells were harvested and homogenized with lysis solution (50 mM Tris-Cl, pH 8.0; 150 mM NaCl; 0.02% sodium azide; 0.1% SDS; 1 μg/ml aprotinin; 1% Nonidet P-40; and 0.5% sodium deoxycholate) containing protease inhibitors (100 μg/ml phenylmethylsulfonyl fluoride, 2 μg/ml aprotinin, 2 μg/ml leupeptin). Supernatant was collected after centrifuging at 12,000 g for 20 min at 4 °C. The BCA protein assay reagent kit (Boster, China) was used for the protein concentration determination. Lysates were resolved by 10% SDS-polyacrylamide gel electrophoresis and transferred to polyvinylidene difluoride (PVDF) membranes. After blocking with 5% nonfat milk, blots were probed with APOC3 antibody (Santa Cruz Biotechnology, USA, sc-50377, lot#3423) and incubated with a peroxidase-conjugated secondary antibody. Bands were visualized by enhanced chemiluminescence reagents (Pierce Chemical, Rockford, IL) and quantified by densitometry.

### Statistical analysis

Statistical analyses were performed with SPSS 13.0 (SPSS Inc, Chicago, Ill) for Windows (Microsoft Corp, Redmond, Wash). Haploview version 4.1 was used to calculate linkage disequilibrium (LD). Deviations of genotype frequency from the Hardy-Weinberg assumption were assessed using χ^2^ test. To test association between the SNPs and lipid traits, we performed multivariate linear regression analysis based on the additive genetic model after adjusting for traditional risk factors. Differences of quantitative variables between groups were analyzed using the Student t-test. The relative luciferase activities of the APOC3 gene 3′-UTR containing either rs4225G or rs4225T were compared using One-way ANOVA method. The plasma APOC3 levels among subjects carrying different genotypes of variant rs4225 were compared using the Kruskal–Wallis test, because the distribution of APOC3 plasma levels is skewed non-normally. The Bonferroni correction method was applied for correction of multiple testing. The case-control association was compared using logistic regression analysis based on the different genetic models with adjustment of traditional risk factors in CHD patients.

All biostatistics calculations were performed using Prism (GraphPad). Comparisons were performed by paired or unpaired t tests, with P < 0.05 considered significant. Comparisons among multiple conditions were performed by ANOVA followed by post hoc t tests. Data are expressed as mean ± SEM of n experiments. All probability values were 2-sided, and p < 0.05 was considered significant.

## Additional Information

**How to cite this article**: Hu, S.-L. *et al*. An APOC3 3′ UTR variant associated with plasma triglycerides levels and coronary heart disease by creating a functional miR-4271 binding site. *Sci. Rep.* **6**, 32700; doi: 10.1038/srep32700 (2016).

## Supplementary Material

Supplementary Information

## Figures and Tables

**Table 1:** Characteristics of APOC3 variants identified by Sanger sequencing.

Gene Position^a^	dbSNP ID^b^	Gene Region	Maj>Min^c^	MAF
chr11:116700775	rs12721090	intron1	C/T	0.082
chr11:116700777	rs618354	intron1	G/C	0.082
chr11:116700785	rs11827682	intron1	C/T	0.082
chr11:116700860	rs734104	intron1	T/C	0.368
chr11:116701028	c.-13-258G > A	intron1	G/A	0.001
chr11:116701146	c.-13-140C > A	intron1	C/A	0.001
chr11:116701122	rs2070669	intron1	G/C	0.475
chr11:116701153	rs2070668	intron1	T/G	0.4
chr11:116701535	rs4520	G34G	T/C	0.391
chr11:116703540	c.240G > A	K80K	G/A	0.001
chr11:116703634	c.*34T > C	3′UTR	T/C	0.003
chr11:116703640	rs5128	3′UTR	C/G	0.196
chr11:116703671	rs4225	3′UTR	G/T	0.185

All variants had at least a 90% genotype call rate and were in Hardy-Weinberg equilibrium (P > 0.05).

^a^Base pair position is based on NCBI GRCh37;

^b^Polymorphisms are numbered relative to transcription start site; The unknown coding variants are called according to the amino-acid position and/or substitution, and the unknown 3′UTR variants are called according to the HGVS nomenclature guidelines (http://www.hgvs.org/mutnomen/).

^c^With major allele given first followed by minor allele; MAF: Minor allele frequency.

**Table 2:** Association of rs4225 Genotype With plasma TG levels.

The general population	Rs4225			Beta (SE)	Trend* p Value	Beta (SE)	Variance Explained (%)	Trend# p Value
	GG(N)	GT(N)	TT(N)					
TG	1891	953	138	−0.09 (0.032)	0.003	−0.024 (0.020)	6.2	0.03
HDL	1891	953	138	0.008 (0.011)	0.65	0.002 (0.007)	4.1	0.84
LDL	1891	953	138	−0.008 (0.025)	0.67	−0.001 (0.01)	3.9	0.94
TC	1891	953	138	−0.023 (0.031)	0.2	0.001 (0.01)	3.2	0.98

^*^Analysis was adjusted for sex and age.

^#^Analysis was adjusted for sex, age, smoking, hypertension, and body mass index.

**Table 3:** Association Between rs4225 Variant with CHD.

SNP rs ID	Function	Population	MAF	*P*_*allele*_	MM	Mm	mm	Model	Crude ORs (95% CI)	Adjusted	Adjusted ORs (95% CI)	
(M > m)					n, (%)	n, (%)	n, (%)			*P*_*value*_		
rs4225	3′-UTR	Control	0.21	0.009	1891	953	138		1.00		1.00	
(G > T)		CHD	0.19		1750	774	103	Additive	0.81(0.62–1.05)	0.18	0.85 (0.68–1.21)	
								Dominant	0.83 (0.64–1.07)	0.21	0.86 (0.71–1.16)	
								Recessive	0.86 (0.78–0.96)	0.009	0.89 (0.77–0.98)	

CHD, Coronary Heart Disease; M, major allele; m, minor allele; MAF, minor allele frequency.

P allele value of allele and Crude odds ratio (95% confidence interval) were determined by a 95% two-sided χ^2^ test, CHD versus controls.

Adjusted ORs (95% CI) and adjusted P-value were obtained with multivariate unconditional logistic regression analysis by adjusting for sex, age, smoking, hypertension, hyperlipidemia, diabetes and body mass index.

**Table 4:** Baseline Characteristics of the study Samples.

Characteristics	The sequencing population	The general population	The CAD population
	Controls (n = 400)	Controls (n = 2982)	Cases (n = 2627)
Age, yrs	59.3 ± 10.5	58.5 ± 10.3	60.0 ± 10.5
Men, %	63.0	55.4	55.3
BMI, kg/m^2^	22.4 ± 2.8	23.2 ± 3.2	21.4 ± 2.5*
SBP, mm Hg	136.8 ± 16.4	138.7 ± 24.4	144.5 ± 21.3*
DBP, mm Hg	81.9 ± 12.7	81.1 ± 12.7	80.4 ± 13.4
Hypertension, %	0	417 (13.98)	1640(62.4)*
Diabetes, %	0	105 (3.52)	450(17.1)*
Hyperlipidemia, %	0	88 (2.95)	357(13.6)*
Smokers, %	0	889 (29.8)	1550(59.0)*
TC (mmol/L)	4.08 ± 0.59	4.31 ± 1.61	4.93 ± 0.97*
HDL (mmol/L)	1.31 ± 0.30	1.46 ± 0.35	2.76 ± 0.79*
LDL (mmol/L)	1.23 ± 0.55	1.12 ± 2.55	2.51 ± 1.03*
TG (mmol/L)	1.05 ± 0.31	1.46 ± 0.99	1.86 ± 1.96*

BMI indicates body mass index; SBP, systolic blood pressure; DBP, diastolic blood pressure;

Values are expressed as mean  ±  SD unless otherwise noted;

^&^test for differences between cases and controls. *p < 0.05. *p < 0.01.
