# Supplementary material for: An APOC3 3′UTR variant associated with plasma triglycerides levels and coronary heart disease by creating a functional miR-4271 binding site
Source: Sci Rep. 2016 Sep 14;6:32700. doi: 10.1038/srep32700 (PMC5021972; doi:10.1038/srep32700)

**Supplementary Materials**

**An APOC3 3’UTR variant associated with plasma triglycerides levels and coronary heart disease by creating a functional miR-4271 binding site**

Sen-Lin Hu, MD, Guang-Lin Cui, MD, PhD, Jin Huang MD, Jian-Gang Jiang, MD, PhD, and Dao-Wen Wang, MD, PhD

From the Institute of Hypertension and Department of Internal Medicine, division of cardiology, Tongji Hospital, Tongji Medical College, Huazhong University of Science and Technology (S.-L.H., G.-L.C., J.H.,J.-G.J., D.-W.W.), Wuhan 430030, China

Corresponding Authors:

Dao Wen Wang, MD, PhD

Departments of Internal Medicine, division of cardiology and Institute of Hypertension

Tongji Hospital, Tongji Medical College

Huazhong University of Science & Technology

1095# Jiefang Ave., Wuhan 430030 People’s Rep. of China

Tel. & Fax: 86-27-8366-3280

Email: [dwwang@tjh.tjmu.edu.cn](mailto:dwwang@tjh.tjmu.edu.cn)

Guang-Lin Cui, MD, PhD

Institute of Hypertension and Department of Internal Medicine, Division of Cardiology,

Tongji Hospital, Tongji Medical College,

Huazhong University of Science and Technology,

Wuhan 430030, People’s Rep. of China

Email: [cuiguanglin@tjh.tjmu.edu.cn](mailto:cuiguanglin@tjh.tjmu.edu.cn)

| **Supplementary Table 1. Angiographic Characteristics of rs4225 Patients** | | | |
| --- | --- | --- | --- |
|  | **rs4225** | |  |
| **Variable** | **GG+GT (2524)** | **TT (103)** | **p value** |
| Coronary artery disease,% |  |  |  |
| LAD | 1795 (71.1) | 70 (68.0) | 0.774 |
| LCX | 1017 (40.3) | 44 (42.7) | 0.751 |
| RCA | 1121 (44.4) | 51 (49.5) | 0.534 |
| More than 1 vessel treated,% | 1567 (62.1) | 55 (53.4) | 0.375 |
| Multivessel disease, % | 1580 (62.6) | 50 (48.5) | 0.146 |

| **Supplementary Table 2. Primers used for PCR and sequencing** | | | |
| --- | --- | --- | --- |
| **Gene Name** | **Primer Coverage** | **Forward Primer Sequence** | **Reverse Primer Sequence** |
| APOC3 | Exon1 | GGACAGCAGCGTGGACTCAG | CTGTGTAGCTTTGGGCAAGTGAC |
|  | Exon2 | TTGATGTTCAGTCTGGTGGGTTTT | TGGCAGGGGGGATGGG |
|  | Exon3 | CCCCTTCTGAGAGCCCGTAT | CCGCAGCAGCCTGACAAA |
|  | Exon4 | CTGGAGATGGCAGGGTTTGA | ATGGCACACTTGCGACGG |
|  | 3'UTR | GGCTGGGTGACCGATGG | AGGGTGATTCCTACCTTAGAGATGA |

| **Supplementary Table 3. Sequence of probes and primers sets** | | | |
| --- | --- | --- | --- |
| **Rs ID** | **primer(5'→3')** | **probe(5'→3')** | **allele** |
| rs4225 | F 5'-AGCTCCTTGGGTCCTGCAAT-3' | 5' FAM-CAGGGCTTCCCCT-MGB 3' | T |
|  | R 5'- AGCACTGAGAATACTGTCCCTTTTAAG-3' | 5' VIC-AGGGCTGCCCCTG-MGB 3' | G |

FAM, 6-carboxyfluorescein; HEX, hexachloro-6-carboxyfluorescein. MGB indicates MGB probe.

| **Supplementary Table 4. Baseline Characteristics of the ELISA study Samples** | |
| --- | --- |
| **Characteristics** | **Controls (n=164)** |
| Age, yrs | 58.6±11.3 |
| Men, % | 63.0 |
| BMI, kg/m^2^ | 22.4±2.8 |
| SBP, mm Hg | 139.8±24.4 |
| DBP, mm Hg | 81.3±13.3 |
| Hypertension, % | 0 |
| Diabetes, % | 0 |
| Hyperlipidemia, % | 0 |
| Smokers, % | 0 |
| TC(mmol/L) | 4.79±0.98 |
| HDL(mmol/L) | 1.46±0.35 |
| LDL(mmol/L) | 2.23±0.77 |
| TG (mmol/L) | 1.42±0.27 |
| BMI indicates body mass index; SBP, systolic blood pressure; DBP, diastolic blood pressure; | |
| Values are expressed as mean ± SD unless otherwise noted. | |

| **Supplementary Table 5. Sequence of miRNA-4271 inhibitor(s)** | |
| --- | --- |
| **miR ID** | **Sequence** |
| hsa-miR-4271 | 5'-gggggaagaaaaggugggg-3'  5'-ccccaccuuuucuuccccc-3' |
| hsa-miR-4271 inhibitor |

**Supplementary Fig 1.** Linkage disequilibrium (LD) structure and haplotype blocks of the APOC3 gene. LD (D’) for identified polymorphisms in APOC3, as generated by Haploview 4.0 from the genotype data of 384 random controls. Haplotype blocks derived from these genotypes using the solid spine LD setting are outlined in black.


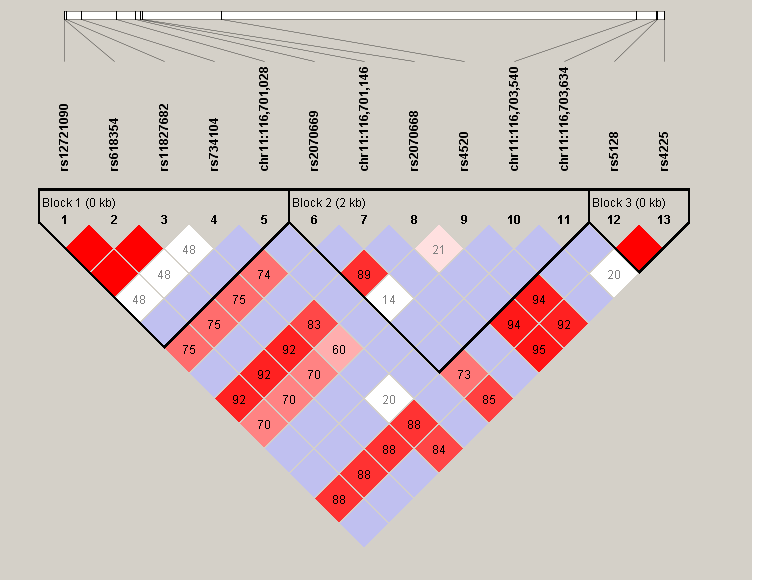


**Supplementary Figure 2.** The interaction between miR-4271 and APOC3 3’ UTR using reporter gene assay in 293T cells. Luciferase plasmid contains pMIR-G or pMIR-T was cotransfected with negative control miRNA (miR-NC), miR-4271 or miR 4271 inhibitor. For each transfection, at least six replicate assays were performed. Luciferase activity was normalized by Renilla luciferase activity for each sample.

**
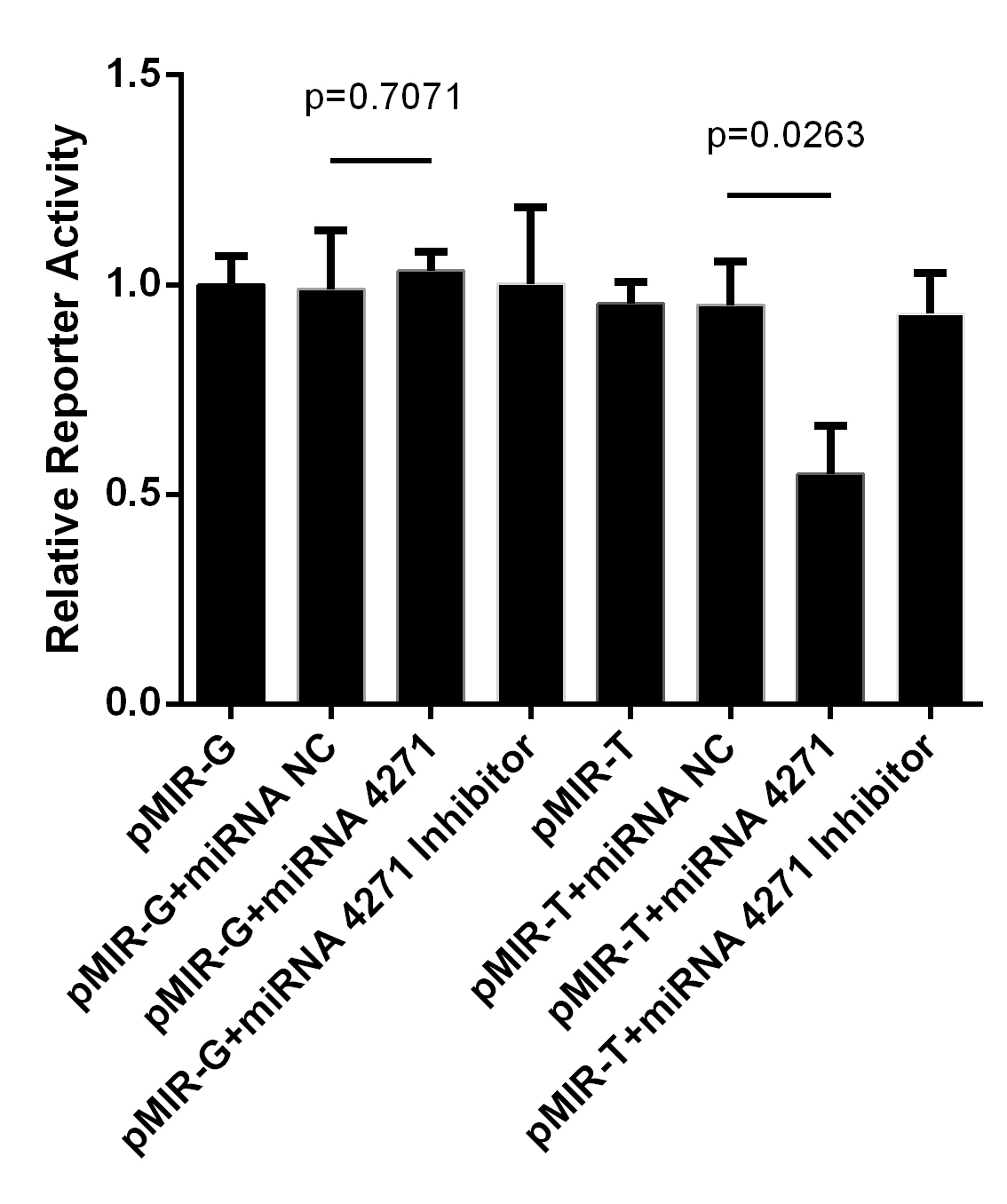
**

**Supplementary Fig 3.** DNA sequences for HepG2 and 97L cell lines. The TT genotype for HepG2 cell (a) and GG genotype for 97L cells (b).

**
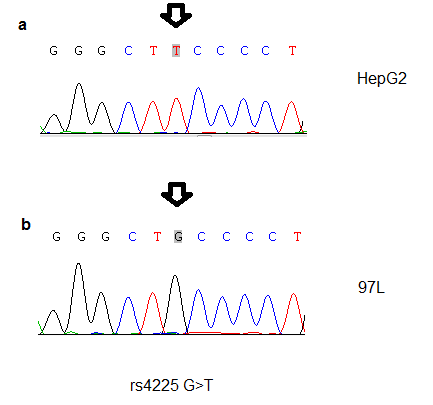
**

**Supplementary Fig 4.** miR-4271 showed no effect on regulation of APOC3 expression analyzed by western blotting in homozygote genotype 97L cells. Differences in expression levels across the different treatment groups were compared by ANOVA. The probability value was corrected for multiple comparisons. The data are presented as mean ± SEM from three independent experiments analyzed in six replicates.


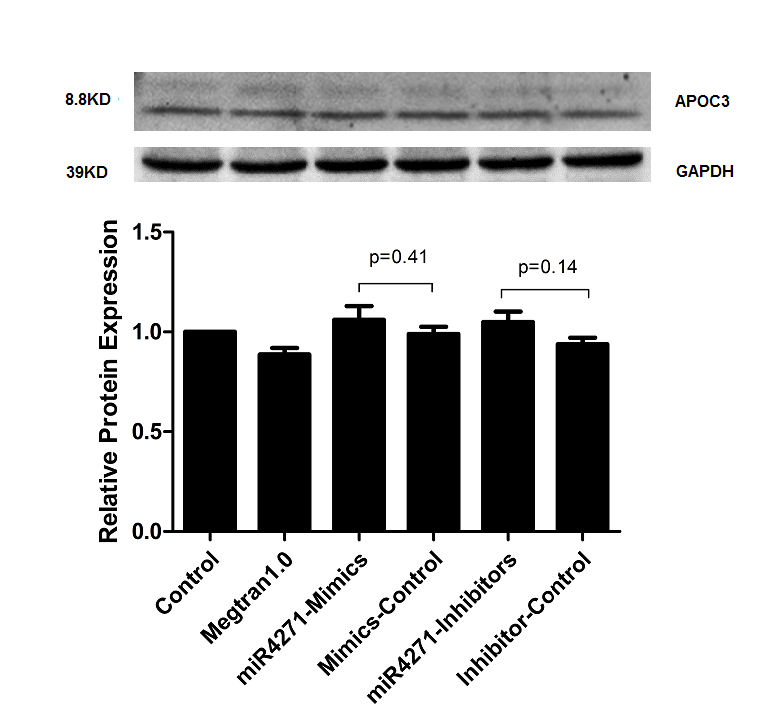

Supplement: Supplementary Information [file srep32700-s1.doc]
